# Potential Molecular Mechanisms of *Zhibai Dihuang Wan* in Systemic Lupus Erythematosus Based on Network Biology

**DOI:** 10.1155/2020/7842179

**Published:** 2020-04-13

**Authors:** Zi Yang, Rui-fei Xie, Min-hong Zhong, Guan-qun Xie, Yong-sheng Fan, Ting Zhao

**Affiliations:** ^1^The First Affiliated College of Medicine, Zhejiang Chinese Medical University, Hangzhou 310000, China; ^2^Hangzhou Cancer Institute, Hangzhou Cancer Hospital, Hangzhou 310000, China; ^3^College of Basic Medical Sciences, Zhejiang Chinese Medical University, Hangzhou 310000, China

## Abstract

Systemic lupus erythematosus (SLE) is a refractory autoimmune disease. *Zhibai Dihuang Wan* (ZDW) has frequently been used for treating SLE in China and been proved to have a prominent role in decreasing SLE patients' morality rate. However, the active substances in ZDW and the molecular mechanisms of ZDW in SLE remain unclear. This study identified the bioactive compounds and delineated the molecular targets and potential pathways of ZDW by using a network biology approach. First, we collected putative targets of ZDW based on TCMSP, GeneCards, and STITCH databases and built a network containing the interactions between the putative targets of ZDW and known therapeutic targets of SLE. Then, the key hubs were imported to DAVID Bioinformatics Resources 6.7 to perform gene ontology biological process (GOBP) and pathway enrichment analysis. A total of 95 nodes including 73 putative targets of ZDW were determined as major hubs in terms of their node degree. The results of GOBP and pathway enrichment analysis indicated that putative targets of ZDW mostly were involved in various pathways associated with inflammatory response and apoptosis. More importantly, eleven putative targets of ZDW (CASP3, BCL2, BAX, CYCS, NFKB1, NFKBIA, IL-6, IL-1β, PTGS2, CCL2, and TNF-*α*) were recognized as active factors involved in the main biological functions of treatment, implying the underlying mechanisms of ZDW acting on SLE. This study provides novel insights into the mechanisms of ZDW in SLE, from the molecular level to the pathway level.

## 1. Introduction

Systemic lupus erythematosus (SLE) is a prototype autoimmune disease with a strong genetic component, characterized by hyperactive T and B cells, autoantibody production, immune complex deposition, and multiorgan damage [1]. Given the disease's potential to cause severe and widespread organ damage, patients with SLE have to take on a big financial burden and cope with intangible loss [2]. Currently, therapeutic agents for SLE patients include hydroxychloroquine, glucocorticoids, immunosuppressive drugs, and biological agents [3]. While the remission of the disease's symptoms and signs is the main goal of SLE patient management, the minimization of drug side effects, which include, for instance, bone marrow, hepatic and pulmonary disorders, Cushing's syndrome, and oculopathy, is an important aspect as well [4, 5]. Consequently, traditional Chinese medicine (TCM), with clinical application for thousands of years, is an attractive alternative to improve SLE patients' survival quality with fewer side effects [6]. *Zhibai Dihuang Wan* (ZDW, or Anemarrhena, Phellodendron, and Rehmannia Pill) has frequently been used for treating SLE in China and been proved to have a prominent role in decreasing SLE patients' morality rate [6–8]. ZDW is composed of eight Chinese herbs, namely, Anemarrhenae Rhizoma (AR, *zhī mŭ*), Phellodendri Chinensis Cortex (PCC, *huáng băi*), Rehmanniae Radix Praeparata (RRP, *shú dì huáng*), Rhizoma Dioscoreae (RD, *shān yào*), *Cornus officinalis* (CO, *shān zhū yú*), poria cocos (PC, *fú líng*), *Alisma orientale* (AO, *zé xiè*), and Cortex Moutan (CM, *mŭ dān pí*). ZDW exerts many effects, such as enriching yin, subduing fire, and returning fire to its source. Meanwhile, patterns of yin deficiency and fire exuberant are the most typical in TCM's view on SLE [9]. Due to its remarkable therapeutic effects, ZDW has been made into patent pills, which are approved by the China Food and Drug Administration (approval number Z41021904) and are widely available in China. However, the active compounds and the molecular mechanisms of ZDW in the treatment of SLE remain to be elucidated. Network biology can help clarify targets and mechanisms of TCM and translate an experience-based theory into an evidence-based one [10].

In the present study, we, respectively, collected the information of targets from active ingredients in ZDW and related targets of SLE from several databases for the first time. Network construction and topological structural analysis were established, which may provide a basis for a more comprehensive understanding of the action mechanisms of ZDW in the treatment of SLE.

## 2. Materials and Methods

### 2.1. Active Compound Screening

The entire compound data of ZDW were retrieved from the TCM Systems Pharmacology Database and Analysis Platform (TCMSP, http://ibts.hkbu.edu.hk/LSP/tcmsp.php) [11]. The active compounds from ZDW were first filtered by integrating oral bioavailability (OB) and drug likeness (DL). Based on literature and suggestions in TCMSP, we selected OB ≥ 30% and DL ≥ 0.18 as a screening threshold. The compounds conforming to both standards mentioned above will be preserved for further analysis.

### 2.2. Putative Target Prediction of the Compounds within ZDW

The integrative efficacy of the compounds in ZDW was determined by analyzing the compounds and target interactions obtained from the GeneCards database (https://www.genecards.org, v4.10.0) and the STITCH database (http://stitch.embl.de/, ver. 5.0) with the species limited as “*Homo sapiens*” and the condition of high confidence (0.700) [12, 13]. GeneCards Database is a human gene database that provides comprehensive information on all annotated and predicted human genes with a collection of gene-centric data from approximately 150 web sources. The target genes with relevance score ≥ 1 were selected for further study. STITCH is a database to explore known and predicted interactions between chemicals and proteins, covering 9,643,763 proteins from 2,031 organisms. Duplications and unified names were removed from the targets obtained from the two tools.

### 2.3. SLE-Associated Targets

There are 3 sources used for predicting SLE-associated targets. The first batch of genes associated with SLE were collected from the Online Mendelian Inheritance in Man (OMIM) database (http://www.omim.org/, updated in May, 2019), which provides over 1,500 relevant genes assigned to the known diseases [14]. The second source was the GeneCards database. The targets that belonged to the protein coding category with relevance score ≥ 1 in the GeneCards database were selected for further study. Lastly, we collected the target genes of known drugs for SLE from the Drugbank. Duplicate names were removed from the targets obtained from these three tools.

### 2.4. Network Construction

To comprehensively understand the molecular mechanisms of the herbs in ZDW, the compound-target network was constructed by linking the active compounds with their potential targets by Cytoscape 3.7.1 [15]. Given that the main function in a TCM formula is mostly determined by the chief herbs, we focused on studying the hub nodes in the network. A node would be defined as a hub when the degree of the node was more than twice the median degree of all the nodes in the same network [16]. Hub targets and central compounds were obtained for further analysis.

### 2.5. Gene Ontology Enrichment Analysis

The gene ontology (GO) biological process (BP) was analyzed with the limitation of “*Homo sapiens*” to further validate whether the hub targets are indeed a match for SLE. The GO enrichment analysis was performed using the functional annotation tool of DAVID Bioinformatics Resources 6.7(https://david-d.ncifcrf.gov/) [17]. The terms with Expression Analysis Systematic Explorer scores of ≤0.05 were selected for functional annotation clustering.

### 2.6. Pathway Enrichment Analysis

To comprehensively understand the molecular mechanisms of ZDW, the dominating target-pathway network was constructed using Cytoscape 3.7.1. The significant pathways were identified by performing enrichment analysis of the proteins by using DAVID Bioinformatics Resources 6.7 and were extracted from KEGG (Kyoto Encyclopaedia of Genes and Genomes, http://www.kegg.jp). Then, we analyzed the top 10 significant pathways and their related targets to elucidate the molecular mechanisms.

## 3. Results

### 3.1. Identification of Active Compounds in ZDW

A total of 729 compounds in ZDW were retrieved from TCMSP, namely, 81 in AR, 140 in PCC, 71 in RD, 226 in CO, 76 in RRP, 34 in PC, 46 in AO, and 55 in CM. 126 compounds met the criteria of OB ≥ 30% and DL ≥ 0.18 simultaneously, accounting for 17% in ZDW (Table 1).

### 3.2. Target Prediction

Putative targets of active compounds in ZDW were merged after they had been collected from the GeneCards and STITCH databases. There were 207 putative targets for AR, 547 for PCC, 64 for RRP, 260 for RD, 472 for CO, 92 for PC, 71 for AO, and 546 for CM. After eliminating the overlapping targets in the eight herbs, we considered 1,075 targets, which pertained to the herbs in ZDW as putative targets. 41 corresponding compounds in ZDW were active compounds. Detailed information about active compounds is provided in supplementary detail. 2,758 gene symbols were collected by the GeneCards, OMIM, and Drugbank databases as SLE-associated targets. 466 overlapped targets of ZDW and SLE were obtained as potential intersections, which corresponds with 39 active compounds. Detailed information about overlapped targets with Uniprot ID [18] is provided in supplementary data 2.

### 3.3. Compound-Target Network and Analysis

**Figure 1:**
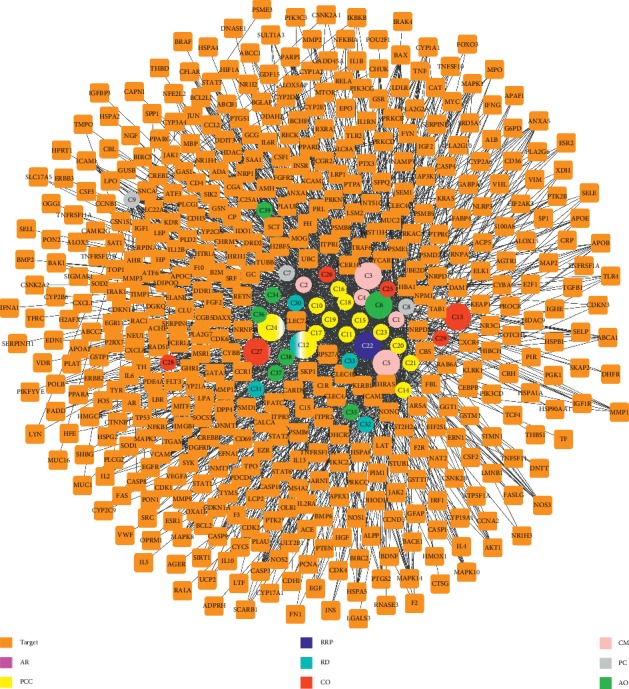
CT network. The multicolored circles represent compounds of different herbs (purple for AR, yellow for PCC, dark blue for RRP, cyan for CO, red for RD, pink for CM, grey for PC, and green for AO) and orange squares represent the targets of each compound. One target can have multiple compounds and vice versa. AR, Anemarrhenae Rhizoma; PCC, Phellodendri Chinensis Cortex; RRP, Rehmanniae Radix Praeparata; RD, Rhizoma Dioscoreae; CO, *Cornus officinalis*; PC, poria cocos; AO, *Alisma orientale*; CM, Cortex Moutan.

To determine the relationship between the 39 active compounds of ZDW with their putative targets, a compound-target (CT) network was built (Figure 1) first. The CT network was constructed using the 466 drug targets, which revealed 505 nodes and 1,200 edges. In such a network, nodes with a degree greater than twice the median are considered key nodes; accordingly, 73 hub targets and 22 central compounds were obtained for further study.

Among 73 hub targets, ZDW exhibited great action on CASP3 (degree = 16), which has been identified as the executioner of apoptosis and the key enzyme of the apoptosis cascade. Similarly, ZDW also strongly affected CASP9 (degree = 11) and CASP8 (degree = 10), suggestive of its contribution to apoptosis. Beyond that ZDW could influence BCL2 (degree = 12) and BAX (degree = 12) to regulate apoptosis. Meanwhile, ZDW also has the potential to act on the MAP kinase family, which participates in the cellular processes like proliferation, differentiation, and development, such as MAPK1 (degree = 12), MAPK14 (degree = 11), and MAPK8 (degree = 9).

The top 5 of the 22 central compounds in ZDW are quercetin (degree = 228), ethyl oleate (degree = 134), (+)-catechin (degree = 129), berberine (degree = 96), and kaempferol (degree = 76).

### 3.4. GO Enrichment Analysis for Targets

**Figure 2:**
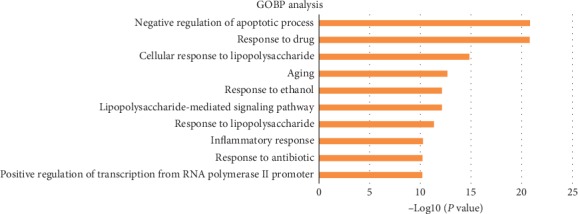
Gene ontology biological process analysis.

A GO enrichment analysis was performed using DAVID Bioinformatics Resources 6.7 to clarify the mechanism of ZDW's main action in SLE. 73 hub targets obtained from the CT network were included in the GO enrichment analysis.  Figure 2 lists the 10 most significantly enriched GOBP terms (*p* ≤ 0.05). The results revealed that numerous targets are involved in the regulation of apoptotic process, lipopolysaccharide (LPS), and inflammation.

### 3.5. Target-Pathway Network and Analysis

The KEGG pathway enrichment analysis was performed with the 73 hub targets by using the functional annotation tool of DAVID Bioinformatics Resources 6.7. The pathways with *P* value ≤ 0.05 are presented in Table 2. We found ZDW acts on 16 pathways in signal transduction, such as the tumor necrosis factor (TNF) signaling pathway. ZDW could affect the endocrine system, immune system, nervous system, and digestive system in SLE patients. Furthermore, ZDW also regulates other pathways in cellular processes, development, metabolism, and genetic information processing.

**Figure 3:**
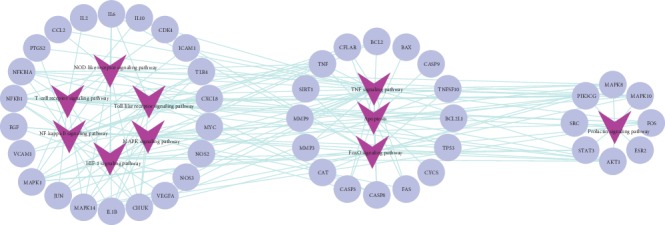
The interaction network between key hubs and the most significant pathways. Round purple blue nodes stand for putative targets of ingredients contained in ZDW as well as the known therapeutic targets for SLE; V-shaped dark purple nodes stand for the most significant pathways based on enrichment analysis of key hubs.

Modularity is a critical measurement for analyzing a network. Nodes highly interconnected within a network usually participate in the same biological modules. In terms of functional distribution, the interaction network of the top 10 significant signaling pathways and corresponding targets was divided into 3 modules. The groups of the main pathways and modules are shown in Figure 3. The maximum module mostly focused on inflammatory response, including the NOD-like receptor signaling pathway, the toll-like receptor (TLR) signaling pathway, the T-cell receptor signaling pathway, the NF-*κ*B pathway, the hypoxia inducible factor (HIF)-1 signaling pathway, and the mitogen-activated protein kinase (MAPK) signaling pathway. The medium module is related to apoptosis, including the TNF signaling pathway, apoptosis, and the Forkhead Box O (FoxO) signaling pathway. The minimum module concerned prolactin (PRL).

According to Table 2, the toll-like receptor signaling pathway, the NOD-like receptor signaling pathway, and the T-cell receptor signaling pathway are categorized in the immune system. Immune response can be considered the most critical mechanism of SLE. The pathogenesis of SLE could be characterized by a complex network of alterations affecting both adaptive and innate immunity [19]. In the present study, the shared targets of ZDW and SLE focused more on the inflammation aspect of immune response, such as the targets related to the NF-*κ*B and MAPK signaling pathway. Suppression of the NF-*κ*B and MAPK signaling pathway in SLE could reduce proinflammatory cytokine production [20, 21]. Hypoxia is closely related to inflammation as well. Extensive crosstalk exists between the HIF pathway and the NF-*κ*B pathway. The HIF pathway has been identified as a possible therapeutic target for diseases including chronic inflammation, infection, and autoimmunity [22].

TNF signaling pathway, which has a leading position among the 10 pathways, plays a crucial role in SLE immunopathogenesis, as it can activate the prosurvival NF-*κ*B and MAPK signaling pathway and induce apoptosis and necroptosis [23]. Apoptosis, as the core pathway in this module, comprises both the intrinsic and extrinsic caspase pathways with the involvement of the shared targets of SLE and ZDW. FoxO proteins also exert great influence on the relationship between the regulation of immune system activity and the induction of apoptotic pathways [24].

The minimum module is centered on the PRL signaling pathway. PRL has an antiapoptotic effect, enhances proliferative response to antigens and mitogens, and enhances the production of immunoglobulins and autoantibodies [25]. It has been demonstrated that hyperprolactinemia is associated with the Systemic Lupus Erythematosus Disease Activity Index (SLEDAI) and stimulates the production of autoantibodies. This proves that PRL plays an important role in SLE [26].

## 4. Discussion

**Figure 4:**
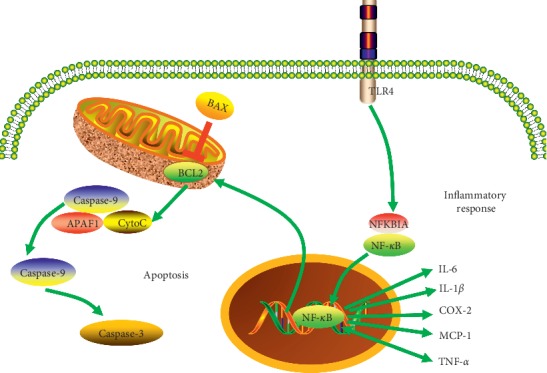
Illustration of crucial biological progress caused by putative targets and known therapeutic targets for SLE. BCL2, B-cell CLL/lymphoma 2; BAX, BCL2-associated X; CYCS, cytochrome C somatic; APAF1, apoptotic peptidase activating factor 1; NF-*κ*B, nuclear factor-kappa B; NFKBIA, NF-kappa-B inhibitor alpha, IL-6, interleukin-6; IL-1β, interleukin 1 beta; PTGS2, prostaglandin-endoperoxide synthase 2; CCL2, C-C motif chemokine ligand 2; TNF-*α*, tumor necrosis factor-alpha.

SLE is an autoimmune disease mediated by pathogenic autoantibodies directed against nucleoprotein complexes [27]. Therapeutic agents for SLE, including hydroxychloroquine, glucocorticoids, and immunosuppressive drugs, are limited due to their adverse effects [28]. ZDW has been clinically proved effective in treating SLE and been approved by the China Food and Drug Administration. However, the compounds in ZDW are complicated and the action mechanisms in SLE patients remain unclear. In the present study, we managed to determine 22 central compounds in ZDW which may exert great influence on SLE treatment. Quercetin could ameliorate the lupus nephritis (LN) symptoms and has renoprotective effects in the LN mice model [29, 30]. (+)-Catechin is recognized as one of the main compounds in green tea polyphenols, which might be a new approach to manage the skin manifestation of SLE [31]. Kaempferol could prevent the progress of autoimmune diseases like SLE by enhancing the Treg cell-suppressive function [32]. The hub targets of ZDW associated with SLE are closely related to apoptosis and inflammatory response, according to the results. Moreover, the literature presents eleven hub targets with a definite relationship with extracts of ZDW's components, including CASP3 (caspase 3), BCL2 (B-cell CLL/lymphoma 2), BAX (BCL2-associated X), CYCS (cytochrome C somatic), NF-*κ*B, NFKBIA (NF-kappa-B inhibitor alpha), IL(interleukin)-6, TLR4, IL-1β, PTGS2 (prostaglandin-endoperoxide synthase 2), CCL2(C-C motif chemokine ligand 2), and TNF-*α* (tumor necrosis factor-alpha). The main biological processes related with certain ZDW regulation are shown in Figure 4.

Patients with SLE often display a deficiency in clearing apoptotic cells. The accumulation of postapoptotic remnants and fragments derived from secondary necrotic cells in the presence of autoantibodies against apoptotic cells or adaptor molecules obliges their pathological elimination and maintains autoinflammation, which is responsible for the initiation of SLE [33]. Thus, apoptosis is essential for the development of SLE. Results of hub targets' analysis found that SLE and ZDW shared a total of 38 targets related to apoptosis, accounting for a substantial part of the entire hub targets. Leukocyte apoptosis is significantly higher in patients with SLE and correlates well with the levels of several autoantibodies [34]. Caspase activation is critical in the entire process of apoptosis, and CASP3 appears to be the major executioner caspase during the demolition phase of apoptosis [35, 36]. An inhibition of the expression of CASP3 could suppress the process of apoptosis, decreasing the postapoptotic remnants and fragments. The extract of PCC could attenuate CASP3 activation and has protective effects against neuronal apoptosis [37]. BCL2 family members, like BCL2 and BAX, could regulate apoptosis via the intrinsic pathway [38]. Suppression of the antiapoptotic members or activation of the proapoptotic members of the BCL2 family leads to altered mitochondrial membrane permeability resulting in a release of CYCS into the cytosol. Quercetin, the shared compound of PCC and CM, could downregulate proapoptotic proteins including BAX, CYCS, and CASP3 and upregulate the antiapoptotic protein, BCL2, via inhibiting the activation of the NF-*κ*B signaling way [39]. Extract of PCC can markedly elevate the ratio of the protein and mRNA levels of BCL2/BAX, while remarkably decrease the release of CYCS and the protein and mRNA expression of CASP3 [40]. Extract of PC also can decrease the expression of apoptotic protein BAX and activity of CASP3, while enhancing the expression of antiapoptotic protein BCL2 [41]. More importantly, a decrease of BCL-2/BAX protein ratio and activation of caspase-3 and 9 are closely related with increased TLR4 and NF-*κ*B expressions. This suggests that the TLR4/NF-*κ*B signaling pathway could regulate apoptosis [42]. Interestingly, ZDW in its entirety has been shown to significantly reduce apoptotic cells via increasing caspase-3 cleavage and improve renal function in gentamicin-induced injury in mice [43]. However, the upstream mechanism of ZDW's negative apoptosis regulation remains to be explored.

NF-*κ*B, as a key transcription factor involved in the regulation of immune responses and apoptosis, could promote the inflammatory responses in the body. NFKBIA, known as I-kappa-B-alpha (I-*κ*B*α*), which inhibits the activity of dimeric NF-kappa-B/REL complexes, has been reported as a target for glucocorticoid-mediated immunosuppression [44]. It has been demonstrated that the extract of PC can reduce the production of inflammatory mediators by suppressing the NF-*κ*B signaling pathway [45]. Although all TLRs could lead to NF-*κ*B activation, we found that ZDW might be more sensitive to an LPS-induced TLR4/NF-*κ*B signaling pathway. LPS could bind the CD14/TLR4/MD2 receptor complex in many cell types, especially in monocytes, dendritic cells, macrophages, and B cells, so as to promote the secretion of proinflammatory cytokines [46]. As a receptor for LPS, the upregulation of TLR4 is responsible for the sustained activation of the cells involved in autoantibody production [47, 48]. The extract of RD has the effect of downregulating the protein level of TLR4 and suppressing the increased levels of inflammatory cytokines [49]. Cytokines can be produced when the NF-*κ*B signaling pathway is activated during the progression of a direct autoantibody-mediated tissue injury and the deposition of complement-fixing immune complexes, inducing chronic inflammatory response. Importantly, the activation of NF-*κ*B is critically responsible for the secretion of cytokines including, IL-6, IL-1β, PTGS2, CCL2, and TNF-*α* [50, 51]. IL-1β and IL-6 belong to the IL family as inflammatory cytokines and could impact on SLEDAI as inflammatory mediators in the active stage of disease. PTGS2, known as cyclooxygenase-2 (COX-2), is involved in the inflammation process in LN [52]. Extract of RD could decrease proinflammatory cytokines such as IL-1β, IL-6, and COX-2 by suppressing the NF-*κ*B signaling pathway [53]. Furthermore, one finding is that the extract of CM has anti-inflammatory effects through the inhibition of COX-2 expression by suppressing the phosphorylation of I-*κ*B*α* and the activation of NF-*κ*B [54]. CCL2, known as monocyte chemoattractant protein-1(MCP-1), a key mediator in inflammatory processes, has a diagnostic value as a specific marker for SLE diagnosis [55]. The component of CM, terpene glycoside, could reduce the proinflammatory molecules IL-6 and MCP-1 expressions [56]. TNF*α*, can not only be activated by canonical NF-*κ*B and MAPK signaling playing a role in inflammation, but can also participate in the derivation of inflammatory responses [57]. The extract of CO could attenuate TNF-*α*-induced NF-*κ*B p65 translocation and suppress the expression levels of MCP-1 induced by TNF-*α* [58]. In summary, ZDW may exert autoantibody elimination by regulating apoptosis-related mechanisms and anti-inflammation function by inhibiting the TLR4/NF-*κ*B signaling pathway and decreasing proinflammatory cytokines in SLE.

## 5. Conclusions

TCM, one of the most important parts of complementary and alternative medicine, markedly contributes to the therapeutic action of autoimmune diseases. This study uses a scientific approach to holistically elucidate that the pharmacological mechanisms of ZDW in the treatment of SLE may be associated with its involvement in apoptosis suppression and anti-inflammation. Among these crucial biological functions, eleven targets were identified as key active factors involved in the main biological processes with validated evidence. However, to comprehensively understand the mechanism of ZDW, further experimental research needs to be undertaken to validate if ZDW treats SLE through this mechanism as a formula.

## Figures and Tables

**Table 1:** Compounds in ZDW that satisfied the criteria of OB ≥ 30%, DL ≥ 0.18, and both.

Herbs	Total	OB ≥ 30%	DL ≥ 0.18	OB ≥ 30% and DL ≥ 0.18
AR	81	28(35%)	48(59%)	15(19%)
PCC	140	86(61%)	70(50%)	37(26%)
RD	71	41(58%)	37(52%)	16(23%)
CO	226	102(45%)	57(25%)	20(9%)
RRP	76	25(33%)	41(54%)	2(3%)
PC	34	18(53%)	25(74%)	15(44%)
AO	46	23(50%)	26(57%)	10(22%)
CM	55	26(47%)	36(65%)	11(20%)
ZDW	729	349(48%)	340(47%)	126(17%)

AR, Anemarrhenae Rhizoma; PCC, Phellodendri Chinensis Cortex; RRP, Rehmanniae Radix Praeparata; RD, Rhizoma Dioscoreae; CO, *Cornus officinalis*; PC, poria cocos; AO, *Alisma orientale*; CM, Cortex Moutan; ZDW, Anemarrhena, Phellodendron, and Rehmannia Pill; OB, oral bioavailability; DL, drug likeness.

**Table 2:** Signal pathway of ZDW's targets.

Pathway class	Pathway name	ZDW's targets on pathway
Signal transduction	TNF signaling pathway	PIK3CG, ICAM1, CFLAR, IL-6, TNF, CCL2, PTGS2, MMP9, NFKBIA, NFKB1, MAPK10, MMP3, AKT1, VCAM1, MAPK1, FOS, CASP3, JUN, MAPK14, CASP8, IL1B, MAPK8, FAS, CHUK
	NF-kappa B signaling pathway	VCAM1, ICAM1, CFLAR, TNF, PTGS2, BCL2, CXCL8, NFKBIA, IL1B, NFKB1, TLR4, BCL2L1, CHUK
	FoxO signaling pathway	PIK3CG, IL-6, MAPK10, SIRT1, IL10, STAT3, AKT1, MAPK1, TNFSF10, MAPK14, MAPK8, CAT, EGF, CHUK
	HIF-1 signaling pathway	PIK3CG, AKT1, MAPK1, IL-6, BCL2, VEGFA, NFKB1, TLR4, NOS3, NOS2, EGF, STAT3
	MAPK signaling pathway	TNF, TP53, NFKB1, MAPK10, AKT1, MAPK1, FOS, CASP3, JUN, MAPK14, IL1B, MAPK8, FAS, EGF, MYC, CHUK
	Sphingolipid signaling pathway	PIK3CG, AKT1, MAPK1, TNF, MAPK14, BAX, BCL2, TP53, NFKB1, MAPK8, NOS3, MAPK10
	VEGF signaling pathway	PIK3CG, AKT1, MAPK1, CASP9, PTGS2, MAPK14, VEGFA, NOS3, SRC
	PI3K-Akt signaling pathway	PIK3CG, IL-6, TP53, NFKB1, TLR4, BCL2L1, CDK4, AKT1, MAPK1, CASP9, BCL2, VEGFA, NOS3, EGF, MYC, CHUK, IL2
	ErbB signaling pathway	PIK3CG, AKT1, MAPK1, JUN, MAPK8, MAPK10, EGF, MYC, SRC
	Ras signaling pathway	PIK3CG, AKT1, MAPK1, VEGFA, NFKB1, MAPK8, BCL2L1, MAPK10, EGF, CHUK
	Jak-STAT signaling pathway	PIK3CG, AKT1, IL-6, BCL2L1, MYC, IL10, STAT3, IL2
	cAMP signaling pathway	PIK3CG, AKT1, MAPK1, FOS, JUN, NFKBIA, NFKB1, MAPK8, MAPK10
	Rap1 signaling pathway	PIK3CG, AKT1, MAPK1, MAPK14, VEGFA, EGF, SRC
	mTOR signaling pathway	PIK3CG, AKT1, MAPK1, TNF
	AMPK signaling pathway	PIK3CG, AKT1, HMGCR, PPARG, SIRT1
	Wnt signaling pathway	JUN, TP53, MAPK8, MAPK10, MYC

Endocrine system	Prolactin signaling pathway	PIK3CG, AKT1, MAPK1, FOS, MAPK14, NFKB1, MAPK8, ESR2, MAPK10, SRC, STAT3
	Estrogen signaling pathway	PIK3CG, AKT1, MAPK1, FOS, JUN, MMP9, NOS3, ESR2, MMP2, SRC
	Insulin resistance	PIK3CG, AKT1, IL-6, TNF, NFKBIA, NFKB1, MAPK8, NOS3, MAPK10, STAT3
	Adipocytokine signaling pathway	AKT1, TNF, NFKBIA, NFKB1, MAPK8, MAPK10, CHUK, STAT3
	GnRH signaling pathway	MAPK1, PTK2B, MAPK14, JUN, MAPK8, MAPK10, MMP2, SRC
	Progesterone-mediated oocyte maturation	PIK3CG, AKT1, MAPK1, CDK1, MAPK14, MAPK8, MAPK10
	Thyroid hormone signaling pathway	PIK3CG, AKT1, MAPK1, CASP9, TP53, MYC, SRC
	Oxytocin signaling pathway	MAPK1, FOS, PTGS2, JUN, NOS3, SRC
	Insulin signaling pathway	PIK3CG, AKT1, MAPK1, MAPK8, MAPK10

Immune system	Toll-like receptor signaling pathway	PIK3CG, IL-6, TNF, CXCL8, NFKBIA, NFKB1, TLR4, MAPK10, AKT1, MAPK1, FOS, JUN, MAPK14, CASP8, IL1B, MAPK8, CHUK
	NOD-like receptor signaling pathway	IL-6, TNF, CCL2, NFKBIA, CXCL8, NFKB1, MAPK10, MAPK1, MAPK14, CASP8, IL1B, MAPK8, CHUK
	T-cell receptor signaling pathway	PIK3CG, AKT1, MAPK1, FOS, TNF, MAPK14, JUN, NFKBIA, NFKB1, CDK4, IL10, CHUK, IL2
	RIG-I-like receptor signaling pathway	TNF, MAPK14, CASP8, CXCL8, NFKBIA, NFKB1, MAPK8, MAPK10, CHUK
	B-cell receptor signaling pathway	PIK3CG, AKT1, MAPK1, FOS, JUN, NFKBIA, NFKB1, CHUK
	Chemokine signaling pathway	PIK3CG, AKT1, MAPK1, CCL2, PTK2B, CXCL8, NFKBIA, NFKB1, SRC, CHUK, STAT3
	Fc epsilon RI signaling pathway	PIK3CG, AKT1, MAPK1, TNF, MAPK14, MAPK8, MAPK10
	Natural killer cell-mediated cytotoxicity	PIK3CG, ICAM1, MAPK1, CASP3, TNFSF10, TNF, PTK2B, FAS
	Cytosolic DNA-sensing pathway	IL-6, NFKBIA, IL1B, NFKB1, CHUK
	Platelet activation	PIK3CG, AKT1, MAPK1, MAPK14, NOS3, SRC

Nervous system	Neurotrophin signaling pathway	PIK3CG, AKT1, MAPK1, MAPK14, JUN, BAX, BCL2, TP53, NFKBIA, NFKB1, MAPK8, MAPK10
	Retrograde endocannabinoid signaling	MAPK1, PTGS2, MAPK14, MAPK8, MAPK10
	Cholinergic synapse	PIK3CG, AKT1, MAPK1, FOS, BCL2
	Inflammatory mediator regulation of TRP channels	PIK3CG, MAPK14, IL1B, MAPK8, MAPK10, SRC

Digestive system	Bile secretion	LDLR, HMGCR, ABCB1, NR1H4

Cellular processes	Apoptosis	PIK3CG, CFLAR, TNF, CYCS, TP53, NFKBIA, NFKB1, BCL2L1, AKT1, TNFSF10, CASP3, CASP9, BAX, BCL2, CASP8, FAS, CHUK
	p53 signaling pathway	CDK1, CASP3, CASP9, BAX, CYCS, CASP8, TP53, FAS, CDK4
	Signaling pathways regulating pluripotency of stem cells	PIK3CG, AKT1, MAPK1, MAPK14, MYC, STAT3
	Cytokine-cytokine receptor interaction	IL-6, TNFSF10, TNF, CCL2, CXCL8, IL1B, FAS, IL10, IL2

Development	Osteoclast differentiation	PIK3CG, TNF, PPARG, NFKBIA, NFKB1, MAPK10, AKT1, MAPK1, FOS, MAPK14, JUN, IL1B, MAPK8, CHUK
Metabolism	Metabolism of xenobiotics by cytochrome P450	CYP3A4, CYP1A1, CYP2D6, CYP1A2
Genetic information processing	Protein processing in endoplasmic reticulum	BAX, BCL2, MAPK8, HSPA5, MAPK10, NFE2L2

## Data Availability

The data used to support the findings of this study are included within the supplementary information files.

## Supplementary Materials

Supplementary Materials The descriptions for supplementary data documents are as follows: supplementary data 1 comprises the active compounds' information in Zhibai Dihuang Wan, including molecule name, oral bioavailability (OB), drug likeness (DL), chemical structure, and affiliated herbs. Supplementary data 2 comprises the overlapped targets of Zhibai Dihuang Wan and systemic lupus erythematosus and their Uniprot ID.
