# Supplementary material for: Potential Molecular Mechanisms of Zhibai Dihuang Wan in Systemic Lupus Erythematosus Based on Network Biology
Source: Evid Based Complement Alternat Med. 2020 Apr 13;2020:7842179. doi: 10.1155/2020/7842179 (PMC7178533; doi:10.1155/2020/7842179)
Supplement: Supplementary Materials — The descriptions for supplementary data documents are as follows: supplementary data 1 comprises the active compounds' information in Zhibai Dihuang Wan, including molecule name, oral bioavailability (OB), drug likeness (DL), chemical structure, and affiliated herbs. Supplementary data 2 comprises the overlapped targets of Zhibai Dihuang Wan and systemic lupus erythematosus and their Uniprot ID. [file 7842179.f1.zip › 7842179.f1/Supplementary data1.docx]

Supplement 1: Information on active compounds in ZDW

| Number | Molecule name | OB | DL | Molecules structure | Herb name |
| --- | --- | --- | --- | --- | --- |
| MOL000492 | (+)-catechin | 54.83 | 0.24 |  | CM |
| MOL007003 | benzoyl paeoniflorin | 31.14 | 0.54 |  | CM |
| MOL000422 | kaempferol | 41.88 | 0.24 |  | CM/AR |
| MOL000211 | Mairin | 55.38 | 0.78 |  | CM |
| MOL000098 | quercetin | 46.43 | 0.28 |  | CM/PCC |
| MOL000359 | sitosterol | 36.91 | 0.75 |  | CM/RRP/CO/AO |
| MOL000283 | Ergosterol peroxide | 40.36 | 0.81 |  | PC |
| MOL000289 | pachymic acid | 33.63 | 0.81 |  | PC |
| MOL000275 | trametenolic acid | 38.71 | 0.80 |  | PC |
| MOL001454 | berberine | 36.86 | 0.78 |  | PCC |
| MOL002894 | berberrubine | 35.74 | 0.73 |  | PCC |
| MOL000358 | beta-sitosterol | 36.91 | 0.75 |  | PCC/CO |
| MOL005438 | campesterol | 37.58 | 0.71 |  | PCC/RD |
| MOL002666 | Chelerythrine | 34.18 | 0.78 |  | PCC |
| MOL001458 | coptisine | 30.67 | 0.86 |  | PCC |
| MOL002651 | Dehydrotanshinone II A | 43.76 | 0.40 |  | PCC |
| MOL002672 | Hericenone H | 39.00 | 0.63 |  | PCC |
| MOL013352 | Obacunone | 43.29 | 0.77 |  | PCC |
| MOL000785 | palmatine | 64.60 | 0.65 |  | PCC |
| MOL002644 | Phellopterin | 40.19 | 0.28 |  | PCC |
| MOL002662 | rutaecarpine | 40.30 | 0.60 |  | PCC |
| MOL000449 | Stigmasterol | 43.83 | 0.76 |  | PCC/AR/RD/PCC/RRP |
| MOL000787 | Fumarine | 59.26 | 0.83 |  | PCC |
| MOL001771 | poriferast-5-en-3beta-ol | 36.91 | 0.75 |  | PCC/CO |
| MOL001736 | (-)-taxifolin | 60.51 | 0.27 |  | RD |
| MOL000953 | CLR | 37.87 | 0.68 |  | RD |
| MOL000546 | diosgenin | 80.88 | 0.81 |  | RD/AR |
| MOL005440 | Isofucosterol | 43.78 | 0.76 |  | RD |
| MOL001559 | piperlonguminine | 30.71 | 0.18 |  | RD |
| MOL001495 | Ethyl linolenate | 46.10 | 0.20 |  | CO |
| MOL002883 | Ethyl oleate (NF) | 32.40 | 0.19 |  | CO |
| MOL005552 | gemin D | 68.83 | 0.56 |  | CO |
| MOL003137 | Leucanthoside | 32.12 | 0.78 |  | CO |
| MOL002464 | 1-Monolinolein | 37.18 | 0.30 |  | AO |
| MOL000831 | Alisol B monoacetate | 35.58 | 0.81 |  | AO |
| MOL000830 | Alisol B | 34.47 | 0.82 |  | AO |
| MOL000856 | alisol C monoacetate | 33.06 | 0.83 |  | AO |
| MOL000854 | alisol C | 32.70 | 0.82 |  | AO |
| MOL000832 | alisol,b,23-acetate | 32.52 | 0.82 |  | AO |
| MOL004528 | Icariin I | 41.58 | 0.61 |  | AR |
| MOL004373 | Anhydroicaritin | 45.41 | 0.44 |  | AR |
